# Lady With the Blue Hair: An Atypical Cause of Myasthenic Crisis

**DOI:** 10.7759/cureus.60186

**Published:** 2024-05-13

**Authors:** Jomaries O Gomez Rosado, Teresa Perez, Kellie N Fusco, Faryal Ahmed, Tianna L Nelson, Taylor A Smith, Hoan Ma, Tye Barber

**Affiliations:** 1 Osteopathic Medicine, Nova Southeastern University Dr. Kiran C. Patel College of Osteopathic Medicine, Fort Lauderdale, USA; 2 Osteopathic Medicine, Edward Via College of Osteopathic Medicine, Broward Health Medical Center, Fort Lauderdale, USA; 3 Family Medicine, Broward Health Medical Center, Fort Lauderdale, USA

**Keywords:** methylchloroisothiazolinone, refractory myasthenia, myasthenia gravis (mg), autoimmune neuromuscular disease, myasthenia gravis triggers, myasthenic crisis, methylisothiazolinone, myasthenia gravis exacerbations

## Abstract

A myasthenic crisis denotes a severe exacerbation of myasthenia gravis, leading a patient to enter a life-threatening state due to progressing muscle weakness that ultimately results in respiratory failure. A crisis can require intubation, mechanical ventilation, and additional critical care to prevent further decompensation and potentially death. Numerous well-documented precipitating factors exist, such as infections, surgery, stress, and various medications. We present the case of a 43-year-old woman recently diagnosed with myasthenia gravis who has experienced two myasthenic crises since diagnosis without evident triggers such as surgery, changes in medication, or infection. Following an unremarkable initial diagnostic test and continued treatment for the crisis, we sought additional information from the patient’s family member at the bedside. We were informed that two weeks prior to both times of crisis with intubation, the patient had dyed her hair blue. The common chemical component in the two different hair dyes used was methylisothiazolinone, which is suspected to have contributed to the exacerbation of the patient's myasthenia gravis. As more evidence for new precipitating factors of myasthenic crises develops, it is crucial for physicians to quickly identify signs and symptoms of a crisis so appropriate intervention can occur in a time-sensitive manner. In addition, myasthenia gravis patients should be made aware to be cautious of precipitating factors of a crisis, including but not limited to new beauty products.

## Introduction

Myasthenia gravis (MG) is a chronic neuromuscular autoimmune disease that is characterized by skeletal muscle weakness and fatigue. The symptoms worsen with physical exertion and improve during periods of relaxation [1]. MG primarily affects middle-aged women and typically manifests a bimodal distribution in the third and sixth decades of life [2,3]. In this autoimmune condition, the body generates antibodies against postsynaptic nicotinic acetylcholine receptors. In rare instances, the body generates antibodies to a muscle-specific protein called muscle-specific kinase (MuSK) antibodies [1,4].

MG is a complex condition that can present with varying degrees of severity. Initial symptomatic manifestations include weakness of the ocular muscles, resulting in ptosis and/or diplopia with generalized muscle weakness. MG can present as either a localized disease primarily affecting the eyes or a generalized disease involving all skeletal muscles [3,5]. Occasionally, it can lead to respiratory insufficiency with extreme weakness in the extremities and bulbar area; this life-threatening state is known as a myasthenic crisis [6]. This crisis presents as progressive muscle weakness requiring intubation and mechanical ventilation due to respiratory muscle failure [7]. It can be precipitated by current infections, surgery, stress, and specific medications such as antimicrobials and anticonvulsants [1,4].

MG disease management involves the use of acetylcholinesterase inhibitors and immunosuppressants. Depending on the severity of the disease, surgical interventions, intubation, mechanical ventilation, plasma exchange, and human intravenous immunoglobulin (IVIG) therapy may be indicated [8,9].

## Case presentation

A 43-year-old African-American female presented to the emergency department due to shortness of breath. Her husband reported that she had felt fatigued, short of breath, and had headaches over the last two weeks. The morning of admission, her husband noted her shortness of breath had worsened and rushed her to the hospital. The patient’s medical history only included MG without known triggers. Three months prior to this admission, she received her initial diagnosis after experiencing a myasthenic crisis; she presented with shortness of breath at a different medical facility. She was intubated for three days and given IVIG and steroids. Her current medications include pyridostigmine 60 mg every 12 hours. Past surgical history included a cholecystectomy. The patient had no known drug allergies, and she rarely consumed alcoholic beverages.

Upon initial evaluation, the patient was delirious with varying levels of awareness. She was not responding to questions or commands. The patient had vibrant blue-colored hair. Pupils were equal, round, and reactive to light. Cardiovascularly, she had a regular rate and rhythm with no murmurs, rubs, or gallops. Respirations were labored. The abdomen was soft, nondistended, and nontender to palpation, with no signs of organomegaly. The patient's vital signs were recorded as follows: temperature, 37.7°C; heart rate, 74 beats per minute; blood pressure, 135/89 mmHg; and oxygen saturation, 84% on bilevel positive airway pressure (BiPAP). As her oxygen levels continued to decrease, a rapid sequence activation was called, and the patient was intubated successfully by the emergency physician without complications. Initial arterial blood gas (ABG) in the emergency department on a 3 L nasal cannula is noted in Table 1.

**Table 1:** Arterial blood gas analysis in the emergency department pH: potential of hydrogen, PCO_2_: partial pressure of carbon dioxide, PO_2_: partial pressure of oxygen, HCO^-3^: bicarbonate

Arterial Blood Gas Analysis	Results	Reference Range
pH	7.16	7.35–7.45
PCO_2_	130 mmHg	35–45 mmHg
PO_2_	178 mmHg	80–99 mmHg
HCO^-3^	35 mEq/L	22–29 mEq/L

The electrocardiogram showed sinus tachycardia, the chest X-ray showed no cardiopulmonary process, and the computed tomography angiography (CTA) showed no evidence of pulmonary emboli. Initial laboratory tests displayed a complete blood count (CBC) within normal limits; remarkable laboratory findings are noted in Table 2. The respiratory panel was all negative. Her vitals were a temperature of 36.7°C, heart rate of 99 beats per minute, and blood pressure of 102/64 mmHg.

**Table 2:** Laboratory findings on admission CRP: C-reactive protein

Parameter	Results	Reference Range
Chloride	90 mmol/L	98-107 mmol/L
Carbon dioxide	>43 mmol/L	22-29 mmol/L
Phosphorus	3.7 mg/dL	2.3–4.7 mg/dL
Magnesium	1.7 mg/dL	1.6–2.6 mg/dL
Troponin	0.18 ng/mL	Less than 0.20 ng/dL
CRP	0.04 mg/dL	Less than 0.3 mg/dL
Procalcitonin	0.02 ng/dL	Less than 0.09 ng/dL
Lactic acid	1.7 mmol/L	0.5–2.2 mmol/L

The patient was transferred to the intensive care unit (ICU), where she was started on a continuous infusion of propofol, norepinephrine, and fentanyl. Additional treatments of methylprednisolone, IVIG, and pyridostigmine were given. Repeat ABG following intubation showed improvements with a pH of 7.38, PCO_2_ of 68 mmHg, PO_2 _of 304 mmHg, and a HCO^-3^ of 35 mEq/L. She continued to get serial ABGs during her hospital stay.

The patient was hospitalized for a total of 13 days, and the course of the stay was noted. On day two, she remained intubated, but her CTA showed a worsening appearance with new basilar airspace disease without evidence of effusion or pneumothorax. She was diagnosed to have pneumonia and started on a short course of antibiotics. On day four, the patient showed improvement, which led to her being extubated and switched to a nasal cannula. However, she decompensated and had to be intubated. She continued her treatment of IVIG, methylprednisolone, and pyridostigmine. On day eight, the patient was extubated and put on BiPAP. She continued to show improvements while transitioning from a high-flow nasal cannula to room air. On day 13, the patient was discharged with home medications and a BiPAP device for nighttime usage. The patient had written instructions for follow-up with specialists.

## Discussion

MG is a difficult illness as there are still so many unknown triggers associated with myasthenic crisis [6]. We believe that, in this case, there was a commonality between this patient’s two exacerbations. On both occasions, the patient dyed her hair with drugstore products, followed by the two-week onset of symptoms. For the initial hospitalization, she dyed her hair red, while for the subsequent admission, it was dyed blue. There is a high possibility that, following dyeing her hair, she was receiving microdoses of the chemical methylisothiazolinone (MIT) until she reached the threshold of crisis. This became obvious to us as the patient’s dye was still leaving residue on her linens two weeks following the initial treatment with the product. The likelihood is that she was self-inoculating and creating a myasthenic crisis.

Several factors contributing to the exacerbation of myasthenic symptoms have been documented in the medical literature. Surprisingly, up to 50% of cases still present triggers that remain unidentified [6]. Recent studies and case reports have pointed to a potential association between myasthenic exacerbations and exposure to certain chemical constituents commonly found in cosmetic products [10]. In the case under current consideration, the most probable trigger for the myasthenic crisis appears to be the utilization of hair dye. This conclusion is drawn from the fact that the patient experienced myasthenic exacerbations on two distinct occasions after using different brands of hair dye. Upon examination of the ingredient lists of these products, it was revealed that they both contained the compounds MIT and methylchloroisothiazolinone (MCI). Given the absence of other discernible factors, there is a possibility that these chemical compounds present in the hair dye might have triggered an immunogenic response, thereby precipitating the myasthenic crisis.

MIT and MCI are cyclic, sulfur-containing organic compounds widely used as preservatives. They are used to control slime-forming bacteria, fungi, and algae in liquid cosmetics, household products, pesticides, water storage, and personal care products [11]. These preservatives are especially common in cosmetics, including bath products, body lotions, makeup, deodorants, skin care products, and hair care products such as hair dye [12]. Despite MIT and MCI having a high prevalence in everyday products, concern for exposure has grown over the past decade. MIT is considered a human sensitizer to the extent that it was named Allergen of the Year in 2013 by the American Contact Dermatitis Society [13]. This led to regulations on the use of MIT in some regions. In the European Union (EU), the mixture of MCI/MIT in leave-on products is banned, and a maximum concentration is restricted to 15 ppm in rinse-off products [14]. Additionally, the EU has prohibited MIT alone in leave-in products and restricted rinse-off products to a concentration of 15 ppm [15,16]. Progress has been made to reduce MIT exposure in the EU, but many other countries, including the United States, have an MIT maximum concentration of 100 ppm [17]. Due to MIT being a strong sensitizer, issues, including local effects and systemic toxicities, continue to arise worldwide [18].

Notably, MIT and MCI have been the subject of concern due to their association with pulmonary damage. In a study examining the implications of MIT and MCI in the context of humidifier disinfectant-associated lung injury (HDLI), it was reported that these disinfectants are the causative agents of fatal lung injury, including interstitial pneumonitis and lung fibrosis in children, pregnant women, and adults [19]. Additionally, MIT has moderate acute toxicity and significant toxicological effects by the oral and inhalation routes. For instance, microscopic lesions in the nasal turbinate from inhalation exposure have been identified [11]. Nonetheless, no previous studies have investigated the relationship between MIT and MCI in the context of the MG crisis. For patients susceptible to a myasthenic crisis with progression to respiratory failure, controlling the exposure to harmful substances can prevent exacerbations and maintain respiratory health.

In a separate study, the adverse effect of MIT on aquatic organisms, planarian flatworms, was explored. The study revealed that even at low concentrations (33 μM and 39 μM), planarians presented with neuromuscular abnormalities that inhibit wound healing and tissue regeneration [20]. This observation bears significance when considering conditions such as MG. While the study primarily focuses on aquatic organisms, it demonstrated that MIT could induce neuromuscular toxicity, which could be relevant to MG, where the neuromuscular junction is a critical site of pathology. However, more research is needed to establish a direct link between MIT exposure and MG exacerbation in humans, as this study primarily deals with planarian models and aquatic toxicity.

## Conclusions

Myasthenic crisis can be precipitated by many factors. In our patient, the possible trigger was believed to be hair dye. We suspect the patient may have become sensitized to MIT, which was found to be an ingredient in the hair dye used prior to both occasions of crisis. Our patient was fortunate to survive a second crisis within a three-month period due to the prompt responsiveness of the physicians in identifying and treating appropriately. While there are many known factors that exacerbated MG, additional ones are still being revealed. Therefore, it is important for patients with MG to be aware of the ingredients in beauty products in an attempt to minimize exposure to possible harmful toxins and prevent future crises.
